# Probing the impact of conventional oil frying on the formation of polycyclic aromatic hydrocarbons in rabbit meat

**DOI:** 10.1002/fsn3.2144

**Published:** 2021-01-21

**Authors:** Rabia Siddique, Ameer Fawad Zahoor, Hamad Ahmad, Faisal Maqbool Zahid, Muhammad Abid, Azhari Siddeeg

**Affiliations:** ^1^ Department of Chemistry Government College University Faisalabad Pakistan; ^2^ Department of Chemistry University of Management and Technology Lahore Lahore Pakistan; ^3^ Department of Statistics Government College University Faisalabad Pakistan; ^4^ Department of Food Engineering Faculty of Engineering University of Gezira Wad Medani Sudan

**Keywords:** different recipes, frying, gas chromatography, mass spectrometry, PAHs

## Abstract

The study estimates, for the first time, the polycyclic aromatic hydrocarbons (PAHs) concentration (pyrene, fluoranthene, phenanthrene, anthracene, fluorene, and naphthalene) in rabbit meat samples. The study explores the effect of frying and the influence of cooking recipe (with or without condiments/additives) on different parts (hind legs, forelegs, and back), on PAH generation. A total of 36 meat samples on different parts from uncooked, cooked, chapli, and seekh kebabs were prepared and characterized by gas chromatography/mass spectrometry (GC/MS). PAHs data in all the samples indicate that cooking recipes (with and without additives/condiments) greatly affected the PAHs concentration. Significant concentrations of phenanthrene, fluoranthene, and naphthalene were formed in all the samples after cooking (frying) but naphthalene was dominant in terms of its concentration formed. A higher concentration of naphthalene was detected in the foreleg (4.56 µg/g) as well as in the hind leg (4.08 µg/g) seekh kebab (with additives), while foreleg chapli kebab (with additives) contained 1.44 µg/g PAH concentration. Frying is the processing methodology that causes the highest impact on PAHs. Contents of anthracene were detected only in the back (raw sample and chapli kebab), foreleg (without additives and seekh kebab), and hind leg (seekh kebab). In all rabbit meat samples, fluorene and pyrene were not identified.

## Graphical abstract

This present study covers the effect of frying on raw materials (hind leg, back, foreleg) and also revealed the effect of condiments on rabbit meat. Statistical analysis of fried samples confirmed that the contents of PAHs were found to be highly significant (*p* <.05) in the comparison between three groups of categories and between one group of samples. Among all the investigated rabbit meat samples, the highest naphthalene concentration was detected in the foreleg and then hind leg seekh kebab. The concentration of naphthalene among different parts of the rabbit was significantly varied due to fat content. Fluorene and pyrene were not observed in all samples. The method of sample preparation, the addition of additives, and a heat source are responsible for the highest PAHs formation. Further study is in the process to find out the new parameters that could overcome the production of PAHs in rabbit meat.

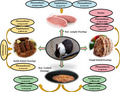

## 1 INTRODUCTION

Polycyclic aromatic hydrocarbons (also termed as polynuclear aromatic hydrocarbons or polyaromatic hydrocarbons) consist of condensed benzene rings without any heteroatom. PAHs comprised of up to four fused benzene rings are known as light polycyclic aromatic hydrocarbons and those composed of more than four aromatic rings are known as heavy polycyclic aromatic hydrocarbons (Mohammadi et al., 2020). PAHs are nonpolar molecules and generated by pyrolysis (burning) or incomplete burning of organic/foodstuff materials such as wood, fossil fuels, tobacco, and trash (Kafouris et al., 2020). They are also present in food materials such as vegetables, fruits, dairy items, tea, coffee, cereal products, and charbroiled meat (Han et al., 2020).

Cooking and processing of food by different domestic cooking practices such as frying, roasting, and grilling over fry pan and charcoal at high temperatures generate various kinds of PAHs (Adeyeye, 2020). At high temperatures, cooking of food *via* pan‐frying, deep‐frying and grilling oil produce adverse substances including, PAHs, particulate matter, carbonyl, and volatile organic compounds (Ghorbani et al., 2020). Oil smoke behaves as the main source for PAHs in kitchens as well as indoor areas (Racovita et al., 2020).

According to SCF (Scientific Committee on Food), JECFA (Expert Committee on Food Additives), FAO/WHO (the Joint Food and Agricultural Organization/ World Health Organization), and USEPA (United State Environmental Protection Agency), 15 PAHs have been considered carcinogenic, mutagenic, and cytotoxic to humans (Authority, 2008; Racovita et al., 2020).

Multistep cleanup procedures and techniques are required for isolation of PAHs including column chromatography, solid‐phase extraction (SPE), microwave‐assisted extraction (MAE), fluid extraction (SFE), gel permeation chromatography (GPC), accelerated solvent extraction (ASE), liquid‐liquid extraction with organic solvents and solid‐phase microextraction (SPME) (Purcaro et al., 2007; Zang et al., 2020). The main analytical procedures, such as HPLC/FLD (high‐performance liquid chromatography with fluorescence detection) (Fazeli et al., 2020; Sun et al., 2020) and GC/MS (Akdoğan & Gürsoy, 2020; Kwok et al., 2020), are employed for quantitative and qualitative evaluation of PAHs in food.

PAHs content in traditional meat dishes is heavily based on thermal treatment of food, fat contents, heat resources, and cooking time (Authority, 2008). Many studies have been made for the estimation of PAHs in meat. The rabbit meat‐eating rate in Asian countries is high and common meat dishes in Asian countries (Pakistan, India, Bangladesh, etc.) such as chapli kebab and seekh kebab are prepared at high temperatures which produce PAHs. However, to our knowledge, no such study has been previously conducted on the formation of PAHs in fried rabbit meat. Previously, our research group has described the determination of PAHs in Aseel chicken (Zahoor et al., 2015) and grilled rabbit meat (SIDDIQUE et al., 2020); herein, we aimed to investigate the formation of PAHs content in commercially fried rabbit dishes with and without additives/condiments *via* indirect heat methodology. Also, PAH analysis was included to evaluate correlations between processed samples with raw samples.

## 2 MATERIALS AND METHODS

### 2.1 Chemicals and reagents

For the preparation of food samples, potassium hydroxide, dichloromethane, methanol, anhydrous sodium sulfate, *n*‐hexane were purchased from Scharlau, while sodium sulfide nonahydrate and HPLC grade acetonitrile were from Sigma‐Aldrich. PAH standards: naphthalene, fluoranthene, and phenanthrene from Fluka, fluorene from Alfa Aesar, pyrene from Hopkins Williams LTD, and anthracene from BDH. Silica gel 60 with 70–230 mesh size was obtained from Merck. The rabbit for sample preparation was purchased from the supermarket of Faisalabad, Pakistan. The 36 samples of meat (foreleg, back, and hind leg) were prepared by frying.

### 2.2 Treatment of sample

For preparation of the sample, thirteen rabbits (approx. 410 g each rabbit weighing) were purchased from local market of Faisalabad, Pakistan. Rabbit meat was washed with water and removed bones and extra fat layers. After drying, the rabbit samples (hind leg, back, and foreleg) were separately homogenized by using a Waring food blender (Milford, MA, USA) for preparing kebabs and kept in the refrigerator. Kebabs were prepared without additives as well as with additives in chapli kebab and seekh kebab recipes. Every meat sample (hind leg, back, and foreleg) including three replicates was examined for PAHs. A list of delicious Asian meat dishes has been presented in Table 1.

**TABLE 1:** Depiction of Asian meat dishes

Samples type	Description
Cooked	**Meat type:** Foreleg, hind leg, and back meat were minced and homogenized
	**Additives:** No additive use
	**Frying conditions:** 10 mints from both sides
	**Samples keeping conditions:** After chilling, samples were packed in transparent nylon bags. These samples were utilized for the cleanup procedure.
Chapli Kebab	**Meat type:** Foreleg, hind leg, and back meat were minced
	**Additives:** Minced meat homogenized and mixed with butter (3.4 g), crushed red pepper (0.24 g), coriander powder (0.23 g), crushed cumin seeds (0.17 g), pomegranate seeds (0.48 g), flour (0.9 g), onion (10 g), tomato (3.54 g), salt (0.05 g) and egg (1 ml).
	**Frying conditions:** Prepared chapli kebabs were fried over the flame for 10 min in a pan
	**Samples keeping conditions:** After chilling, samples were packaged in transparent nylon bags. These samples were utilized for the cleanup procedure.
Seekh kebab	**Meat type:** Foreleg, hind leg, and back were minced
	**Additives:** Minced meat homogenized with onion (4.48 g), green chili (1.14 g), ginger (0.26 g), garlic (0.26 g), coriander leaves (0.3 g), mint (0.3 g), mustard powder (0.08 g), salt (0.27 g) and black pepper powder (0.15 g) for 30 min.
	**Cooking condition:** The seekh kebabs were fried for 10 min.
	**Samples keeping conditions:** After chilling, samples were packaged in transparent nylon bags. These samples were utilized for the cleanup procedure.

### 2.3 Preparation of sample

For meat sample preparation, the methodology described by Chung et al. (2011) with minor modifications was used. A convenient procedure for the determination of PAHs in meat contents comprises saponification of 30 g homogenized sample of meat in 500 ml flat‐bottom flask along with 2 g of sodium sulfide nonahydrate and 2 M KOH solution (100 ml) in CH_3_OH‐H_2_O mixture (9:1, v/v) and was allowed to reflux in a water bath for 2 hr at 70°C. After cooling, *n*‐hexane (100 ml) was added through the condenser with the subsequent addition of cold water (100 ml) after 15 min. The sample mixture was kept overnight in dark. After 24 hr, 60 ml *n*‐hexane was added to the sample extracted the *n*‐hexane layer from the aqueous layer, and repeated the extraction procedure with 2 × 30 ml *n*‐hexane followed by drying over Na_2_SO_4_. The organic phase was evaporated with rotavapor at 35°C, and the obtained concentrate (2 ml) was purified by column chromatography. The column was packed with a slurry of silica (added 20 g of silica gel to approximately 40 ml *n*‐hexane), and then, concentrate was poured on the column. Elution was carried out using 50 ml of *n*‐hexane then passed 8 ml of 3:1 (v/v) *n*‐hexane‐dichloromethane ratio through the column which was concentrated approximately 1 ml by using rotavapor. The concentrate was filtered by a microporous syringe (0.45 µm) and stored in the refrigerator at −20°C for GC/MS analysis (Kazerouni et al., 2001).

### 2.4 GC‐MS analysis

The quantitative analysis of meat samples was performed by using an Agilent brand (7890B) Gas Chromatograph coupled with Mass Spectrometer (5977A) (Kao et al., 2014). Separation of PAHs was done on a column DB‐5MS with length: 30 m × 0.25 mm ID and thickness: 0.25 µm. 1 µl sample was inserted in splitless mode. Highly pure Helium gas (purity > 99.995%) was utilized as a carrier gas with flow rate of 1 ml/min applied and other settings for GC‐MS include: injector temperature 290°C; GC oven program 80^o^C/min, rate 25^o^C/min‐260^o^C for 1 min, at 10^o^C/min‐300^o^C for 6.3 min and detector temperature: 150°C‐ion source and 230°C‐quadrupole. Chromatogram run time optimized at 25 min. The minimum limit of detection for each sample throughout this study was 0.009 µg/g. Identification of PAHs was done using the calibration method. Stock (concentrated) solutions of fluoranthene, naphthalene, pyrene, anthracene, phenanthrene, and fluorene were prepared. 100 µg/ml concentrated solution was prepared by dilution of 100 µg PAH in 1 ml acetonitrile solvent. 20 µg/ml of working solution was prepared by taking a concentrated solution (1 ml) in 4 ml acetonitrile. These calibration curves were made using the standard solution.

### 2.5 Statistical analysis

Two‐way ANOVA was used to compare different categories of rabbit and meat recipes. Every meat sample (hind leg, back, and foreleg) including three replicates were examined for PAHs. The analysis of PAHs data was performed with Minitab 13.2 software.

## 3 RESULTS AND DISCUSSION

**FIGURE 1:**
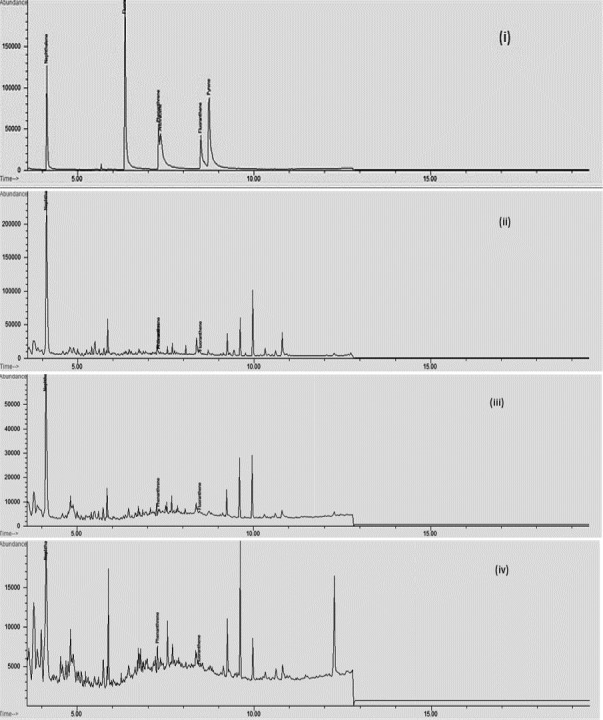
GC‐MS chromatograms (i) Mixed standards (ii) Foreleg seekh kebab (iii) Hind leg chapli kebab (iv) Cooked back sample

Identification of PAHs was achieved from GC‐MS chromatograms which are presented in Figure 1. Data obtained from the quantitative GC‐MS study of PAHs for every food sample expressed in µg/g have been summarized in Table 2. Results indicated that the naphthalene content was significantly (*p *≤ .05) higher in all prepared samples. Evaluated naphthalene concentration in raw samples exhibited following increasing order: back > hind leg > foreleg, while fluoranthene and phenanthrene were significantly (*p* ≤ .05) lower than naphthalene. Raw back samples of rabbits also contained a very small amount of anthracene (0.01 µg/g). These results are reliable with Kao et al. (2014) study who also examined the PAHs (fluoranthene, naphthalene, phenanthrene, and fluorene) concentration in raw chicken and stated that raw sample can be contaminated from polluted air, water, or soil. However, after shallow frying of raw samples for 10 min, the naphthalene concentration in all raw cooked samples was significantly (*p* ≤ .05) higher. Hence, fat molecules have greatly affected PAHs formation during thermal cooking (Saito et al., 2014). The foreleg cooked meat (without condiments) contained the highest concentration of naphthalene while the content of naphthalene was 0.55 µg/g in the hind leg cooked sample and 0.36 µg/g in the back sample (cooked). Because the foreleg portion of the rabbit contains more fat molecules than the hind leg and is followed by the back (Pla et al., 2004). The phenanthrene content in the foreleg portion (cooked) was significantly (*p* ≤ .05) higher in comparison with hind leg and back. The concentration of fluoranthene in fried cooked samples was from 0.04–0.05 µg/g, while 1 PAHs (anthracene) was present in the foreleg (without additives). It has formerly been reported that the generation of PAHs in the sample (meat) is caused by the use of flame. During frying, the heating of cooking oil resulted in the formation of oil fumes that contain aerosol oil droplets, gaseous pollutants, and combustion products. These particles may get adsorbed on meat surface which includes PAHs and thus enhances the PAHs concentration (Humans, 2010).

**TABLE 2:** PAHs content in meat samples determined by GC/MS

Category	Food Samples	Naphthalene (µg/g)	Fluorene (µg/g)	Phenanthrene (µg/g)	Anthracene (µg/g)	Fluoranthene (µg/g)	Pyrene (µg/g)
Uncooked	Hind leg	0.23 ± 0.02_b_^c^	BDL*	0.13 ± 0.01_a_^b^	BDL	0.05 ± 0.003_b_^a^	BDL
	Back	0.15 ± 0.01_a_^c^	BDL	0.19 ± 0.01_c_^d^	0.01 ± 0.01_a_^b^	0.05 ± 0.002_b_^a^	BDL
	Foreleg	0.24 ± 0.01_b_^c^	BDL	0.11 ± 0.01_a_^b^	BDL	0.04 ± 0.002_a_^a^	BDL
Cooked	Hind leg	0.55 ± 0.03_d_^c^	BDL	0.16 ± 0.01_b_^b^	BDL	0.04 ± 0.002_a_^a^	BDL
	Back	0.36 ± 0.02_c_^c^	BDL	0.13 ± 0.01_a_^b^	BDL	0.04 ± 0.002_a_^a^	BDL
	Foreleg	0.86 ± 0.06_e_^c^	BDL	0.21 ± 0.01_cd_^b^	0.01 ± 0.01_a_^b^	0.05 ± 0.003_b_^a^	BDL
Chapli Kebab	Hind leg	1.34 ± 0.08_f_^c^	BDL	0.18 ± 0.01_bc_^b^	BDL	0.06 ± 0.003_c_^a^	BDL
	Back	0.93 ± 0.05_e_^c^	BDL	0.22 ± 0.001_d_^b^	0.01 ± 0.01_a_^b^	0.07 ± 0.003_d_^a^	BDL
	Foreleg	1.44 ± 0.10_f_^c^	BDL	0.19 ± 0.01_c_^b^	BDL	0.05 ± 0.003_b_^a^	BDL
Seekh Kebab	Hind leg	4.08 ± 0.30_g_^d^	BDL	0.23 ± 0.02_d_^c^	0.02 ± 0.01_a_^a^	0.05 ± 0.003_b_^b^	BDL
	Back	1.33 ± 0.07_f_^c^	BDL	0.13 ± 0.01_a_^b^	BDL	0.04 ± 0.002_a_^a^	BDL
	Foreleg	4.56 ± 0.30_g_^d^	BDL	0.21 ± 0.01_cd_^c^	0.02 ± 0.01_a_^a^	0.05 ± 0.007_b_^b^	BDL

* Below detection limit (<0.009 µg/g). Alphabet letters in superscript represent significant differences (*p* ≤ .05) with the same row. Different letters in subscript denote significant (*p* < .05) difference within the similar column.

Formation of PAHs in kebab dishes when the kebabs were prepared with a variety of additives used in chapli kebab and seekh kebab dishes was significantly (*p* ≤ .05) higher than kebabs with no additives. Processing (frying) of meat samples with additives increased the concentration of naphthalene. The naphthalene level was significantly (*p *≤ .05) higher in the foreleg portion and hind leg seekh kebab (4.56 µg/g, 4.08 µg/g). In this recipe, coriander leaves, fresh mint, as well as spices were used. Literature survey revealed that additives affect the concentration of PAHs in meat sample such as processed vegetables contained 0.00197–0.335 µg/g concentration of PAHs (Paris et al., 2018), 0.0496–0.496 µg/g PAHs concentration present in eggs, and 0.0017–0.0217 µg/g PAHs range in butter (Luzardo et al., 2013) and salt exhibited minimum PAHs 0.0003–0.007 µg/g (Kim et al., 2014). The high concentration of PAHs in seekh kebab is connected with additives that cause an increase in PAHs concentration (SIDDIQUE et al., 2020). When compared the results of fried seekh kebab with grilled seekh kebab (SIDDIQUE et al., 2020), the highest PAHs was detected in fried seekh kebab. The results concluded that frying significantly affects the PAH generation.

Moreover, the concentration of naphthalene in the foreleg and hind leg chapli kebab was not significantly (*p* > .05) different, while in back chapli kebab, the level of naphthalene was significantly (*p* ≤ .05) lesser. The concentration of phenanthrene in the back chapli kebab was significantly (*p* ≤ .05) higher as compared to the foreleg and hind leg chapli kebab. Phenanthrene level in seekh kebab of hind leg and foreleg was significantly (*p* > .05) similar. The smaller concentration of fluoranthene in all examined samples was 0.04–0.07 µg/g. The minimum concentration of anthracene was detected only in few samples (with additives); seekh kebab of hind leg and foreleg and chapli kebab of the back. However, the presence of fluorene and pyrene was not observed in investigated samples.

## 4 CONCLUSIONS

For the very first time, the occurrence of PAHs was evaluated in rabbit food: uncooked, cooked without condiments, chapli kebab, and seekh kebab. This present study covers the effect of frying on raw materials (hind leg, back, foreleg) and also revealed the effect of condiments on rabbit meat. Statistical analysis of fried samples confirmed that the contents of PAHs were found to be highly significant (*p* ≤ .05) in the comparison between three groups of categories and between one group of samples. Frying cooking method significantly affects the PAHs formation. Among all the investigated rabbit meat samples, the highest naphthalene concentration was detected in the foreleg and then hind leg seekh kebab. The usage of vegetables increased the PAHs concentration. The concentration of naphthalene among different parts of the rabbit was significantly varied due to fat content. Fluorene and pyrene were not observed in all samples. The method of sample preparation, the addition of additives, and a heat source are responsible for the highest PAHs formation. Further study is in the process to find out the new parameters that could overcome the production of PAHs in rabbit meat.

## 5 ETHICAL REVIEW

This does not involve human or animal modeling.

## CONFLICT OF INTEREST

Authors declare to not have any conflict of interest.
